# Monastrol mimic Biginelli dihydropyrimidinone derivatives: synthesis, cytotoxicity screening against HepG2 and HeLa cell lines and molecular modeling study

**DOI:** 10.1186/2191-2858-2-23

**Published:** 2012-06-12

**Authors:** Uttara Soumyanarayanan, Varadaraj G Bhat, Sidhartha S Kar, Jesil A Mathew

**Affiliations:** 1 Department of Pharmaceutical Chemistry, Manipal College of Pharmaceutical Sciences, Manipal University, Manipal, Karnataka 576104, India; 2 Department of Pharmaceutical Biotechnology, Manipal College of Pharmaceutical Sciences, Manipal University, Manipal, Karnataka 576104, India

**Keywords:** Privileged structures, Amide coupling, Mitotic kinesin, Biginelli reaction

## Abstract

Biginelli dihydropyrimidinone derivatives as structural analogs of monastrol, a known human kinesin Eg5 inhibitor, were synthesized. IC_50_ values of the synthesized compounds against the proliferation of human hepatocellular carcinoma and human epithelial carcinoma cell lines were determined through MTT assay. Molecular docking study gave a clear insight into the structural activity relationship of the compounds in comparison with monastrol.

## **Background**

1,4-Dihydropyrimidinones (DHPMs) comprise of a pyrimidine scaffold having a resemblance with the structures of the nucleic acid bases found in DNA and RNA. Their involvement as bases in nucleic acids has a great significance in drug design. Recent progress in the DHPM class of the anticancer agent monastrol, an inhibitor of human kinesin Eg5 [1,2], has led to the attention for efficient pharmacophore variation of Biginelli DHPMs. Human kinesin Eg5 plays a crucial role in bipolar spindle generation during mitosis, inhibition of which leads to mitotic arrest and subsequent apoptotic cell death [3]. It is therefore considered as one of the promising targets in cancer chemotherapy. Racemic dihydropyrimidinone is reported to be an allosteric inhibitor of Eg5 [4], and unlike taxanes, it is nontoxic to neuron cells [5,6].

Considerable work has also been devoted to gain insights into the structure-activity relationship in the monastrol derivative series [7]. Recently, Dennis Russowsky and coworkers described the differential effects of monastrol, oxo-monastrol and oxygenated analogs on seven human cancer cell lines [8]. However, anticancer activity profile of amide derivatives of dihydropyrimidinones with functional variations at aromatic ring has not been explored so far. This paper describes the synthesis and evaluation of monastrol-related racemic dihydropyrimidinones substituted with privileged structures [9] like pyrrolidine, piperidine and morpholine through an amide linkage. It was speculated that the introduction of cyclic amines through amide linkage at the side chain of the DHPM scaffold could mimic the interactions of the ester group of monastrol and might also provide improved metabolic stability to the moiety. In order to probe the effect of substitutions at the aromatic ring of Biginelli DHPMs on cytotoxicity, halogens were introduced at ortho and para positions. Physicochemical properties and biological activity of thiourea is closely related with urea, which possesses bioisosteric pharmacophore groups. Hence, thiourea motif of the DHPMs has been replaced by urea that may function as a bioisoster. In addition, the molecular docking and virtual physicochemical properties were studied to understand the structural activity relationship of the scaffold.

## **Methods**

### **Chemistry**

#### ***General remarks***

**Scheme 1:**
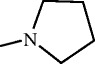
Synthetic scheme for the preparation of compounds 3a to 3 l.

Melting points were taken in capillary tubes, measured in the melting point apparatus and were uncorrected. Infrared spectra were recorded on a Shimadzu FTIR 8310 spectrometer. Nuclear magnetic resonance (NMR) spectra were recorded on Bruker 400/500 MHz spectrometer in CDCl_3_/DMSO-*d*_6_/CD_3_OD. Chemical shifts are reported in parts per million (ppm) from tetramethylsilane with tetramethylsilane as the internal standard. Data reported are as follows: chemical shift, multiplicity as singlet (s), doublet (d), triplet (t), quartet (q), broad singlet (br s) and multiplet (m), and coupling constants (Hz). Mass spectra were obtained on a Shimadzu GCMS-QP5050A. Combustion analyses were performed on a Perkin-Elmer 2400-II analyzer. The progress of the reactions was monitored by thin layer chromatography using F254 silica gel pre-coated sheets (Merck, India). Column chromatography was performed on silica gel (100 to 200 mesh). Solvents for extraction and chromatography were AR/HPLC grade (Scheme 1).

### **General method for the preparation of compounds 1a to 1d**

A solution of arylaldehyde (0.28 mmol), ethyl acetoacetate (0.28 mmol), urea (0.42 mmol) and boric acid (0.056 mmol) in glacial acetic acid (15 mL) was heated at 100°C, stirring for 9 h. Progress of the reaction was monitored by thin layer chromatography (TLC) using hexane:ethyl acetate (4:6) as mobile phase. Then, the reaction mixture was cooled to room temperature, poured into ice-cold water (150 to 200 mL) and stirred for 15 min. The solid precipitate obtained was filtered, washed with ice-cold water and recrystallized with absolute alcohol to afford white solid crystals with a quantitative yield [10].

*Ethyl 4-methyl-2-oxo-6-phenylhexahydropyrimidine-5-carboxylate (1a):* Colorless crystals, Yield 80%. Melting point (Mp.) 200°C to 202°C (Literature (Lit.) [11] Mp. 202°C to 204°C). R_f_ 0.56 (Hexane:ethyl acetate (4:6)). Infra Red Spectra (IR) (KBr) cm^−1^: 3,242 (N-H stretching (str.)), 1,645 (C = O str.). ^1^ H NMR (500 MHz, DMSO-*d*_6_): δ 9.21 (s, 1 H), 7.46 (br s, 1 H), 7.35 to 7.14 (m, 5 H), 5.62 (s, 1 H), 3.95 (q, 2 H), 2.26 (s, 3 H), 0.997 (s, 3 H).

*Ethyl 4-(4-fluorophenyl)-6-methyl-2-oxohexahydropyrimidine-5-carboxylate (1b):* Colorless crystals, Yield 80%. Mp. 178°C to 180°C (Lit. [12] Mp. 175°C to 177°C). R_f_ 0.51 (Hexane:ethyl acetate (4:6)). IR (KBr) cm^−1^: 3,242 (N-H str.), 1,718 (C = O str.), 1,645 (C = O str.). ^1^ H NMR (500 MHz, DMSO-*d*_6_): δ 9.38 (s, 1 H), 7.86 (br s, 1 H), 7.46 to 7.36 (m, 2 H), 7.33 to 7.24 (m, 2 H), 5.56 (s, 1 H), 3.96 (q, 2 H), 2.27 (s, 3 H), 1.03 (s, 3 H).

*Ethyl 4-(4-chlorophenyl)-6-methyl-2-oxohexahydropyrimidine-5-carboxylate (1c):* Colorless crystals, Yield 81%. Mp. 214°C to 216°C. (Lit. [13] Mp. 214°C to 217°C). R_f_ 0.54 (Hexane:ethyl acetate (4:6)). IR (KBr) cm^−1^: 3,350 (N-H str.), 1,637 (C = O str.). ^1^ H NMR (500 MHz, DMSO-*d*_6_): δ 9.57 (s, 1 H), 7.76 (br s, 1 H), 7.43 to 7.38 (m, 2 H), 7.34 to 7.21 (m, 2 H), 5.68 (s, 1 H), 3.87 (q, 2 H), 2.33 (s, 3 H), 0.995 (s, 3 H).

*Ethyl 4-(2-chlorophenyl)-6-methyl-2-oxohexahydropyrimidine-5-carboxylate (1d)*: Colorless crystals, Yield 78%. Mp. 213°C to 215°C. (Lit. [14] Mp. 212.5°C to 214.5°C). R_f_ 0.55 (Hexane:ethyl acetate (4:6)). IR (KBr) cm^−1^: 3,350 (N-H str.), 1,637 (C = O str.). ^1^ H NMR (500 MHz, DMSO-*d*_6_): δ 9.27 (s, 1 H), 7.71 (br s, 1 H), 7.40 (d, 1 H), 7.34 to 7.29 (m, 2 H), 7.28 to 7.25 (m, 1 H), 5.63 (s, 1 H), 3.91 (q, 2 H), 2.3 (s, 3 H), 0.998 (s, 3 H). MS (EI): 295 [M]^+^.

### **General method for the preparation of compounds 2a to 2d**

A solution of compounds 1a to 1d (2 mmol) in 10 mL methanol and NaOH (4 mmol) dissolved in 1 mL water was heated at 60°C to 62°C, stirring for 8 h. Progress of the reaction was monitored by TLC using chloroform:methanol (9:1) as mobile phase. The reaction mixture was cooled and concentrated under vacuum to remove methanol. The residue obtained was then added to 25 mL ice-cold water and was extracted with chloroform (3 × 10 mL) to remove the unreacted ester. The aqueous layer was acidified to pH 2 using 10% *v*/*v* HCl and then extracted with ethyl acetate (3 × 15 mL). The ethyl acetate layer was separated, pooled over anhydrous sodium sulfate and evaporated under vacuum to give the crude acid. The crude acid was purified by column chromatography using chloroform:methanol (95:5) as mobile phase and silica gel (100 to 200 mesh) as stationary phase to afford the pure product [15-17].

*4-Methyl-2-oxo-6-phenylhexahydropyrimidine-5-carboxylic acid (2a)*: Colorless solid, Yield 35%. Mp. 217°C to 219°C. (Lit. [18] Mp. 210°C). R_f_ 0.26 (Chloroform:methanol (9:1)). IR (KBr) cm^−1^: 3,431 (O-H str.), 3,225 (N-H str.), 1,645 (C = O str.), 1,705 (C = O str.).

*4-(4-Fluorophenyl)-6-methyl-2-oxohexahydropyrimidine-5-carboxylic acid (2b)*: Colorless solid, Yield 36%. Mp. 200°C to 202°C. R_f_ 0.28 (Chloroform:methanol (9:1)). IR (KBr) cm^−1^: 3,442 (O-H str.), 3,227 (N-H str.), 1,705 (C = O str.).

*4-(4-Chlorophenyl)-6-methyl-2-oxohexahydropyrimidine-5-carboxylic acid (2c)*: Colorless solid, Yield 38%. Mp. 207°C to 209°C (Lit. [18] Mp. 199°C to 200°C). R_f_ 0.24 (Chloroform:methanol (9:1)). IR (KBr) cm^−1^: 3,439 (O-H str.), 3,238 (N-H str.), 1,643 (C = O str.).

*4-(2-Chlorophenyl)-6-methyl-2-oxohexahydropyrimidine-5-carboxylic acid (2d):* Colorless solid, Yield 37%. Mp. 212°C to 214°C. R_f_ 0.27 (Chloroform:methanol (9:1)). IR (KBr) cm^−1^: 3,444 (O-H str.), 3,238 (N-H str.), 1,708 (C = O str.), 1,645 (C = O str.). ^1^ H NMR (500 MHz, CD_3_OD): δ 7.42 to 7.40 (dd, 2 H, *J* = 7.5 and 1.5 Hz), 7.36 to 7.34 (dd, 1 H, *J* = 8 and 2 Hz), 7.32 to 7.30 (dd, 1 H, *J* = 7.5 and 1.5 Hz), 7.27 to 7.26 (d, 1 H, *J* = 1.5 Hz), 5.83 (s, 1 H), 2.18 (s, 3 H). ESI-MS, 266.5 [M]^+^.

### **General method for the preparation of compounds 3a to 3 l**

A mixture of compounds 2a to 2d was added to tetramethyluronium tetraflouroborate (TBTU) (1.5 eq.) and diisopropylethylamine (2 eq.) in 2 mL dimethylformamide under nitrogen atmosphere. The mixture was stirred for 2 min at 25°C, and then, pyrrolidine/piperidine/morpholine (1.5 eq., 0.068 mL) was added to it. Another 2 equivalents of diisopropylethylamine (DIPEA) was again added to it. The reaction mixture was stirred for 10 min. The reaction was monitored by TLC using chloroform:methanol (9:1). The reaction mixture was poured into 50 mL ice-cold water and was extracted with dichloromethane (3 × 20 mL). The extract was purified by column chromatography using chloroform:methanol (97:3) and silica gel (100 to 200 mesh) as stationary phase to afford the desired product [19,20].

*6-Methyl-4-phenyl-5-(pyrrolidine-1-carbonyl)-3,4-dihydropyrimidin-2(1 H)-one (3a)*: White solid, Yield 81%. Mp. 258°C to 260°C. Rf: 0.46 (Chloroform:methanol (9:1)). IR (KBr) cm^−1^: 3,224, 3,145 (N-H str.), 1,696 (C = O str.), 2,881 (C-H str.). ^13^ C NMR (400 MHz, DMSO-d_6_): δ 166.34 (C = O), 161.83 (C = O), 140.42 to 124.32 (6 C, aromatic), 151.74 (=C-NH), 25.32 to 23.96 (4 C, aliphatic), 15.56 (CH_3_). ^1^ H NMR (400 MHz, CDCl_3_): 7.26 to 7.24 (m, 5 H), 6.38 (br s, 1 H), 5.41 (s, 1 H), 5.11 (s, 1 H), 3.26 to 2.10 (m, 8 H), 1.73 (s, 3 H). MS (EI): 285 [M]^+^, 270 [M-CH_3_]^+^, 214 [M-C_5_H_10_N]^+^, 187 [M-C_6_H_10_NO]^+^, 172 [M-CH_3_-C_6_H_10_NO]^+^, 132 [M-C_9_H_14_N_2_O]^+^.

*6-Methyl-4-phenyl-5-(piperidine-1-carbonyl)-3,4-dihydropyrimidin-2(1 H)-one (3b)*: White solid, Yield 83%. Mp. 266°C to 268°C. R_f_ 0.49 (Chloroform:methanol (9:1)). IR (KBr) cm^−1^: 3,223, 3,095 (N-H str.), 1,681 (C = O str.), 2,858 (C-H str.). ^13^ C NMR (400 MHz, DMSO-d_6_): 165.88 (C = O), 162.28 (C = O), 141.56 to 125.56 (6 C aromatic), 152.26 (=C-NH), 104.48, 56.24 (CH), 26.32 to 23.80 (5 C, aliphatic), 15.78 (CH_3_). ^1^ H NMR (400 MHz, CDCl_3_): δ 8.44 (br s, 1 H), 7.35 (s, 1 H, aromatic H), 7.33 (d, 1 H, *J* = 0.8 Hz), 7.31 (t, 1 H, *J* = 2.4 Hz), δ 7.26 (m, 1 H), 7.19 (d, 1 H, *J* = 0.8 Hz), 7.17 (s, 1 H), 2.95 (s, 4 H), 1.34 (s, 2 H), 1.10 (d, 4 H, *J* = 2 Hz). MS (EI): 299 [M]^+^, 284 [M-CH_3_]^+^, 214 [M-C_5_H_10_N]^+^, 187 [M-C_6_H_10_NO]^+^, 172 [M-CH_3_-C_6_H_10_NO]^+^, 133 [M-C_9_H_14_N_2_O]^+^.

*4-Methyl-5-(morpholine-4-carbonyl)-6-phenyltetrahydropyrimidin-2(1 H)-one (3c)*: White solid, Yield 83%. Mp. 233°C to 234°C. R_f_ 0.52 (Chloroform:methanol (9:1)). IR (KBr) cm^−1^: 3,240 (N-H str.), 1,695 (C = O str.), 2,858 (C-H str.). ^13^ C NMR (400 MHz, DMSO-d_6_): 166.68 (C = O), 162.28 (C = O), 142.28 to 126.23 (6 C, aromatic), 152.38 (=C-NH), 103.76, 56.22 (CH), 30.42 to 26.68 (4 C, aliphatic), 15.32 (CH_3_). ^1^ H NMR (400 MHz, CDCl_3_): δ 7.35 to 7.31(m, 5 H) 7.21 (d, 1 H, *J* = 1.6 Hz) 5.49 (s, 1 H), 5.30 (s, 1 H), 3.22 to 3.05 (m, 6 H) 1.27 to 1.29 (m, 2 H). MS (EI): 301 [M]^+^, 286 [M-CH_3_]^+^, 214 [M-C_4_H_8_NO]^+^, 186 [M-C_5_H_8_NO_2_]^+^, 172[M-CH_3_-C_5_H_8_NO_2_]^+^, 132 [M-C_8_H_12_N_2_O_2_]^+^.

*4-(4-Fluorophenyl)-6-methyl-5-(pyrrolidine-1-carbonyl)-3,4-dihydropyrimidin-2(1 H)-one (3d)*: White solid, Yield 80%. Mp. 242°C to 244°C. R_f_ 0.48 (Chloroform:methanol (9:1)). IR (KBr) cm^−1^: 3,242, 3,142 (N-H str.), 1,689, 1,660 (C = O str.), 2,954 (C-H str.). ^13^ C NMR (400 MHz, DMSO-d_6_): δ 166.57 (C = O), 160.15 (C = O), 162.15 to 114(6 C aromatic), 152.58 (=C-NH), 105.79, 55.6 (CH), 25.11 to 23.80 (4 C, aliphatic), 15.96 (CH_3_). ^1^ H NMR (400 MHz, CDCl_3_): 7.26 to 6.29 (m, 4 H), 6.87 (br s, 1 H), 5.41 (s, 1 H), 5.23 (s, 1 H), 3.26 to 2.59 (m,4 H), 1.67 (s, 3 H), 1.66 to 1.18 (m, 4 H). MS (EI): 303 [M]^+^, 288 [M-CH_3_]^+^, 233 [M-C_4_H_8_NO]^+^, 205 [M-C_5_H_8_NO_2_]^+^, 190 [M-CH_3_-C_5_H_8_NO_2_]^+^, 150 [M-C_8_H_12_N_2_O]^+^.

*4-(4-Fluorophenyl)-6-methyl-5-(piperidine-1-carbonyl)-3,4-dihydropyrimidin-2(1 H)-one (3e)*: White solid, Yield 82%. Mp. 264°C to 266°C. R_f_: 0.53 (Chloroform:methanol (9:1)). IR (KBr) cm^−1^: 3,240 (N-H str), 1,691 (C = O str), 1,658 (amide C = O str). ^13^ C NMR (400 MHz, DMSO-d_6_): δ 165.76 (C = O), 160.52 (C = O), 163.18 to 113.32 (6 C, aromatic), 153.26 (=C-NH), 106.72, 55.4 (CH), 26.52 to 23.20 (5 C, aliphatic), 15.40 (CH_3_). ^1^ H NMR (400 MHz, CDCl_3_): δ 7.55 (s, 1 H), 7.35 to 7.30 (m, 2 H,), 7.05 to 7.01 (m, 2 H), 5.45 (s, 1 H), 5.43 (s, 1 H), 3.86 to 3.04 (m, 4 H), 1.76 (s, 3 H), 1.60 to 1.55 (m, 2 H), 1.46 to 1.41 (m, 2 H), 1.29 to 1.24(m, 2 H). MS (EI): 317 [M]^+^, 302 [M-CH_3_]^+^, 233 [M-C_5_H_10_N]^+^, 222 [M-C_6_H_4_F]^+^, 205 [M-C_6_H_10_NO]^+^, 193 [M-CH_3_-C_6_H_10_NO]^+^, 150 [M-C_9_H_14_N_2_O]^+^.

*4-(4-Fluorophenyl)-6-methyl-5-(morpholine-4-carbonyl)-3, 4-dihydropyrimidin-2(1 H)-one (3f)*: White solid, Yield 82%. Mp. 230°C to 232°C. R_f_ 0.51 (Chloroform:methanol (9:1)). IR (KBr) cm^−1^: 3,248, 3,150 (N-H str.), 1,681, 1,608 (C = O str.), 2,875 (C-H str.). ^13^ C NMR (400 MHz, DMSO-d_6_): δ 166.22 (C = O), 161.36 (C = O), 162.78 to 113.76 (6 C, aromatic), 152.64 (=C-NH), 105.63, 55.28 (CH), 29.22 to 24.46 (4 C, aliphatic), 15.63 (CH_3_). ^1^ H NMR (400 MHz, CDCl_3_): 7.27 to 6.96 (m, 4 H), 6.41 (s,1 H), 5.42 (s, 1 H), 5.10 (s, 1 H), 3.25 to 3.027 (m, 4 H), 1.72 (s, 3 H), 1.48 to 1.18 (m, 4 H). 319 [M]^+^, 303 [M-CH_3_]^+^, 232 [M-C_4_H_8_NO]^+^, 205 [M-C_5_H_8_NO_2_]^+^, 190 [M-CH_3_-C_5_H_8_NO_2_]^+^, 150 [M-C_8_H_12_N_2_O_2_]^+^.

*4-(4-Chlorophenyl)-6-methyl-5-(pyrrolidine-1-carbonyl)-3,4-dihydropyrimidin-2(1 H)-one (3 g):* White solid, Yield 84%. Mp. 260°C to 262°C. R_f_ 0.42 (Chloroform:methanol (9:1)). IR (KBr) cm^−1^: 3,227, 3,151(N-H str.), 1,676, 1,608 (C = O str.), 2,862 (C-H str.). ^13^ C NMR (400 MHz, DMSO-d_6_): δ 165.82 (C = O), 162.28 (C = O), 139.70 to 115.92 (6 C, aromatic), 153.72 (+C-NH), 103.02, 56.75 (CH), 24.78 to 23.20 (4 C, aliphatic), 15.34 (CH_3_). ^1^ H NMR (400 MHz, CDCl_3_): δ 7.55 (br s, 1 H), 7.32 to 7.29 (m, 2 H,), 7.28 to 7.26 (m, 2 H), 5.44 (s, 1 H), 5.46 (s, 1 H), 3.33 (s, 2 H), 3.02 (s, 1 H), 2.68 (s, 1 H), 1.80 (s, 3 H), 1.72 to 1.61 (m, 4 H). ESI-MS: 320 [M]^+^, 249 [M-C_5_H_8_N]^+^, 303 [M-CH_4_]^+^, 207 [M-C_6_H_5_Cl]^+^.

*4-(4-Chlorophenyl)-6-methyl-5-(piperidine-1-carbonyl)-3,4-dihydropyrimidin-2(1 H)-one (3 h)*: White solid, Yield 86%. Mp. 253°C to 255°C. R_f_ 0.48 (Chloroform:methanol (9:1)). IR (KBr) cm^−1^: 3,227, 3,151(N-H str.), 1,676, 1,608 (C = O str.), 2,862 (C-H str.). ^13^ C NMR (400 MHz, DMSO-d_6_): δ 166.25 (C = O), 161.48 (C = O), 138.78 to 116.26 (6 C, aromatic), 152.58 (=C-NH), 103.38, 56.53 (CH), 25.20 to 23.64 (5 C, aliphatic), 15.62 (CH_3_). ^1^ H NMR (400 MHz, CDCl_3_): δ 7.70 (br s, 1 H), 7.32 to 7.24 (m, 4 H), 3.48 to 3.02 (m, 4 H), 1.79 (s, 3 H), 1.66 to 1.25 (m, 6 H). MS (EI): 333[M-1]^+^, 318 [M-CH_3_]^+^, 249[M-C_4_H_8_NO]^+^, 221 [M-C_5_H_8_NO_2_]^+^, 206[M-CH_3_-C_5_H_8_NO_2_]^+^, 166 [M-C_9_H_14_N_20_]^+^.

*4-(4-Chlorophenyl)-6-methyl-5-(morpholine-4-carbonyl)-3,4-dihydropyrimidin-2(1 H)-one (3i)*: White solid, Yield 84%. Mp. 228°C to 230°C. R_f_ 0.44 (Chloroform:methanol (9:1)). IR (KBr) cm^−1^: 3,264, 3,102 (N-H str.), 1,681, 1,624(C = O str.), 2,858 (C-H str.). ^13^ C NMR (400 MHz, DMSO-d_6_): 165.62 (C = O), 162.36 (C = O), 139.28 to 116.44 (6 C, aromatic), 152.76 (=C-NH), 104.28, 56.24 (CH), 29.76 to 24.38 (4 C, aliphatic), 15.78 (CH_3_). ^1^ H NMR (400 MHz, CDCl_3_): 7.28 to 7.20 (m, 4 H), 7.04 (br s, 1 H), 5.41 (s, 1 H), 5.27 (s, 1 H), 3.25 to 3.03 (m, 6 H), 2.22 to 2.10 (m, 2 H), 1.72 (s, 3 H). MS (EI): 335[M]^+^, 320 [M-CH_3_]^+^, 249 [M-C_4_H_8_NO]^+^, 221[M-C_5_H_8_NO_2_]^+^, 207 [M-CH_3_-C_5_H_8_NO_2_]^+^, 166[M-C_8_H_12_N_2_O_2_]^+^.

*4-(2-Chlorophenyl)-6-methyl-5-(pyrrolidine-1-carbonyl)-3, 4-dihydropyrimidin-2(1 H)-one (3j)*: White solid, Yield 83%. Mp. 223°C to 224°C. R_f_ 0.53 (Chloroform:methanol (9:1)). IR (KBr) cm^−1^: 3,221, 3,095 (N-H str.), 1,686 (C = O str.), 2,877 (C-H str.). ^13^ C NMR (400 MHz, DMSO-d_6_): 166.24 (C = O), 162.36 (C = O), 150.25 (=C-NH), 143.66 to 121.54 (6 C, aromatic), 105.65, 53.44 (CH), 26.28 to 23.93 (4 C, aliphatic), 15.65 (CH_3_). ^1^ H NMR (400 MHz, CDCl_3_): δ 7.52 to 7.50 (dd, 1 H, *J* = 7.6 and 1.6 Hz), 7.34 to 7.32 (dd, 1 H, *J* = 7.6 and 1.2 Hz,), 7.30 to 7.23 (dd, 1 H, *J* = 7.6 and 1.6 Hz), 7.22 to 7.20(dd, 1 H, *J* = 7.6 and 1.6 Hz,), 6.91 (br s, 1 H), 5.76 (d, 1 H, *J* = 1.2 Hz), 5.36 (s, 1 H), 3.37 to 3.20 (m, 4 H), 1.89 (d, 4 H, *J* = 0.4 Hz), 1.79 (s, 3 H). MS (EI): 319 [M]^+^, 304 [M-CH_3_]^+^, 284 [M-Cl]^+^, 249 [M-C_4_H_8_N]^+^, 221 [M-C_5_H_8_NO], 206 [M-CH_3_-C_5_H_8_NO]^+^, 166 [M-C_8_H_12_N_2_O]^+^.

*4-(2-Chlorophenyl)-6-methyl-5-(piperidine-1-carbonyl)-3,4-dihydropyrimidin-2(1 H)-one (3 k)*: White solid, Yield 80%. Mp. 240°C to 242°C. R_f_ 0.52 (Chloroform:methanol (9:1)). IR (KBr) cm^−1^: 3,222, 3,109 (N-H str.), 1,685 (C = O str.), 2,949 (C-H str.). ^13^ C NMR (400 MHz, DMSO-d_6_): 166. 38 (C = O), 161.72 (C = O), 141.56 to 122.38 (6 C, aromatic), 151.36 (=C-NH), 104.72, 54.46 (CH), 25.92 to 23.80 (5 C, aliphatic), 15.55 (CH_3_). ^1^ H NMR (400 MHz, CDCl_3_): 7.49 to 7.48 (d, 1 H, *J* = 7.2 Hz), 7.43 (s, 1 H), 7.35 to 7.33 (d, 1 H, *J* = 6.8 Hz), 7.28 (t, 1 H, *J* = 3.6 Hz), 7.24 to 7.22 (dd, 1 H, *J* = 1.6 and 7.6 Hz), 5.74 (s, 1 H), 5.40 (s, 1 H), 3.35 to 3.21 (m, 4 H), 1.84 (s, 3 H), 1.49 to 1.33 (m, 6 H). MS (EI): 333 [M-1]^+^, 318 [M-CH_3_]^+^, 298 [M-Cl]^+^, 249 [M-C_5_H_10_N]^+^, 221 [M-C_6_H_10_NO]^+^, 207 [M-CH_3_-C_6_H_10_NO]^+^.

*4-(2-Chlorophenyl)-6-methyl-5-(morpholine-4-carbonyl)-3,4-dihydropyrimidin-2(1 H)-one (3 l)*: White solid, Yield 80%. Mp. 240°C to 242°C. R_f_ 0.52 (Chloroform:methanol (9:1)). IR (KBr) cm^−1^: 3,240, 3,099 (N-H str.), 1,685(C = O str.), 2,860 (C-H str.). ^13^ C NMR (400 MHz, DMSO-d_6_): 165.42 (C = O), 161.56 (C = O), 140.49 to 121.25 (6 C, aromatic), 153.25 (=C-NH), 104.72, 54.72 (CH), 28.56 to 24.78 (4 C, aliphatic), 15.45 (CH_3_). ^1^ H NMR (400 MHz, CDCl_3_): 7.47 (br s, 1 H), 7.40 to 7.38 (dd, 1 H, *J* = 1.6 and 7.6 Hz), 7.31 to 7.18 (dd, 1 H, *J* = 1.6 and 7.6 Hz), 7.24 to 7.22 (dd, 1 H, *J* = 1.6 and 7.2 Hz), 7.20 to 7.18 (m, 2 H), 3.22 to 2.77 (m, 8 H), 1.77 (s, 3 H). 335[M]^+^, 320 [M-CH_3_]^+^, 249 [M-C_4_H_8_NO]^+^, 221[M-C_5_H_8_NO_2_]^+^, 207 [M-CH_3_-C_5_H_8_NO_2_]^+^, 166[M-C_8_H_12_N_2_O_2_]^+^.

### **Cytotoxicity studies**

In order to assess the *in vitro* cytotoxicity potential of these monastrol mimics, 3-(4,5-dimethylthiazol-2-yl)-2,5-diphenyltetrazolium bromide (MTT) assay on HepG2 and HeLa cell lines was performed. Exponentially growing cell lines were harvested from 25 cm^2^. Tissue culture flasks and a stock cell suspension (1 × 10^5^ cell/mL) were prepared. A 96-well flat-bottom tissue culture plate was seeded with 1 × 10^4^ cells in 0.1 ml of MEM and DMEM mediums supplemented with 10% FBS and allowed to attach for 24 h. Test compounds were prepared just prior to the experiment in 0.5% DMSO and serially diluted with medium to get the working stock of 500, 250, 125 and 62.5 μg/mL solution. After 24 h of incubation, cells were treated with 20 μL of test solutions from the respective top stocks, 80 μL of fresh medium was added, and the cells were incubated for 48 h. The cells in the control group received only the medium containing 0.5% DMSO. Each treatment was performed in triplicates. After the treatment, the drug-containing media was removed and washed with 200 μL of phosphate buffered saline (PBS). To each well of the 96-well plate, 100 μL of MTT reagent (stock, 1 mg/mL in PBS) was added and incubated for 4 h at 37°C. After 4 h of incubation, the plate was inverted on tissue paper to remove the MTT reagent. To solubilize formazan crystals in the wells, 100 μL of 100% DMSO was added to each well. The optical density was measured by an enzyme-linked immunosorbent assay plate reader at 540 nm [21].

### **Molecular modeling**

Molecular modeling studies were performed using a flexible docking method with the Glide version 2010 as described by Yan *et al.*[22] and Garcia-Saez et al. [23]. The X-ray crystal structure of Eg5 complexed with monastrol (Protein Data Bank ID: 1Q0B) was retrieved from the Protein Data Bank. The three-dimensional structures of the DHPM derivatives were constructed with the Chemdraw Ultra 8.0. Energy minimizations were performed by Ligprep using OPLS-2004 force field. The binding affinity of the inhibitors to the protein was then evaluated by the total glide docking energies. The physicochemical properties of the compounds, such as molecular weight, log P, hydrogen bond acceptors/donors, polar surface area (PSA) and number of rotatable bonds of the synthesized compounds, were calculated using QuikPro (Schrodinger-2010).

## **Results and discussion**

### **Chemistry**

The dihydropyrimidinones in this study were prepared by the Biginelli reaction [24] of the substituted benzaldehyde, urea and ethylacetoacetate depicted in Scheme 1. Interestingly, the hydrolysis of DHPM was much slower and stalled at less than 10% conversion. A substantial amount of decarboxylated product was formed during hydrolysis, similar to the earlier reports [7]. Since a simultaneous decarboxylation accompanied the formation of acid, it was found that the temperature and reaction time played a crucial role in the productivity of the reaction.

Since mild hydrolyzing agent could prevent the extent of decarboxylation, lithium hydroxide with different solvent combinations (methanol/THF/dioxane) [25] was used for hydrolysis. The reaction was found to proceed slowly, along with the formation of lesser decarboxylated product, but also resulted in low yields of acid (>20%).

Earlier literature reports of DHPM esters hydrolysis in ethyl acetate and sodium hydroxide (25°C to 27°C, 2 h) [26] also failed to form the acid. The ester did not proceed to form the acid even after refluxing with ethyl acetate for 12 h.

Acid hydrolysis using concentrated HCl and TFA (25°C/reflux) also failed to yield any acid. Refluxing with KOH and ethanol led to decarboxylation due to higher refluxing temperature (around 80°C). These observations propelled us to use a solvent with a lower boiling point.

Studies also reported that refluxing in methanol in the presence of aqueous NaOH (10%) led to hydrolysis and consequent decarboxylation [16,17]. Hence, the optimized method for hydrolysis involved heating the ester in methanol with aqueous NaOH at 60°C to 62°C for 8 h. Prolongation of the reaction time or increase in temperature resulted in decarboxlyation. However, it was observed that higher quantities of NaOH did not affect the decarboxylation process.

The synthesis of compounds 3a to 3 l with a stable amide linker between DHPMs and cyclic amines was achieved via TBTU/DIPEA-promoted coupling reaction, which was quick and high yielding. The physical data of all the synthesized compounds are summarized in Table 1. The mass, proton and carbon NMR spectra of some of the final compounds synthesized can be found in Additional files 1, 2, 3, 4, 5, 6, 7 and 8.

**Table 1:** Structural details and percentage yield of compounds 3a to 3 l

**Compound code**	**R**	**R**_**1**_	**Yield (%)**	**Compound code**	**R**	**R**_**1**_	**Yield (%)**
3a	H	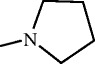	82	3 g	4-Cl		81
3b	H		80	3 h	4-Cl		83
3c	H		82	3i	4-Cl		83
3d	4-F		84	3j	2-Cl		83
3e	4-F		86	3 k	2-Cl		80
3f	4-F		84	3 l	2-Cl		86

R, substitution on the phenyl ring; R_1_, substituted cyclic amine (as depicted in Scheme 1).

### **Cytotoxicity studies**

**Figure 1:**
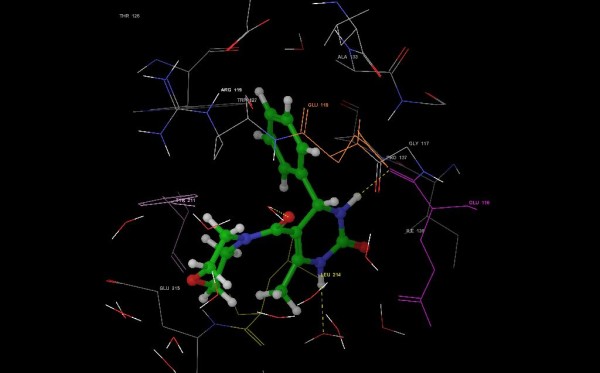
S-monastrol and compound 3 h.

The data obtained from *in vitro* cytotoxicity assay suggest that compounds 3 g and 3 h were found to be the most potent against the HepG2 cell lines with IC_50_ 124.46 and 120.62 μg/mL, respectively. However, most of the compounds exhibited weak activity (IC_50_ 200 μg/mL) against HeLa cell lines. Hence, it was anticipated that substitution of electron-withdrawing substituents, such as chlorine, at the para position may be essential for the ligand-receptor interaction. Structure-activity relationship (SAR) analysis revealed that compounds with weakly basic pyrrolidine and piperidine substitution in the side chain attenuate the anticancer activity. In contrast, morpholine was not tolerated at the side chain of DHPMs and hence resulted in decreased IC_50_. Molecular modeling studies showed the absence of hydrogen bonding interactions with Glu-118, which could be attributed to the missing hydrogen bond donor on the phenyl ring. Hydrogen bonding interactions of water molecules with the oxygen atom of morpholine in compounds 3c, 3f, 3i and 3 l proved to be fatal for anticancer activity. *In vitro* data for these compounds are summarized in Table 2. Figure 1 shows the schematic view of the contacts of S-monastrol and compound 3 h with the residues of Eg5 inhibitor-binding pockets with in 3 Å, and hydrogen bonds are depicted by dashed lines.

**Table 2:** **IC**_**50 **_**of compounds 3a-l by MTT assay**

**Compound code**	**IC**_**50**_^**a**^**(μg/mL)**	
	**HepG2**	**HeLa**
3a	180	216
3b	166	189
3c	207	240
3d	178	229
3e	194	-
3f	269	346
3 g	124	187
3 h	120	217
3i	218	374
3j	192	261
3 k	191	398
3 l	218	374

^a^50% inhibitory concentration. HepG2, human hepatocellular carcinoma; HeLa, human epithelial carcinoma.

### **Structure-activity relationship**

To determine the SAR, functionality modification was carried out on the phenyl ring, while different cyclic amines were introduced at the side chain of DHPMs. Unsubstituted compounds (3a, 3b and 3c) which displayed IC_50_ of 180.92, 166.96 and 207.45 μg/mL and 216, 189 and 240 μg/mL against HePG2 and HeLa cell lines, respectively, served as the template for cytotoxicity potential comparison. Replacement of the protons in compounds 3a, 3b and 3c with fluorine at the para position of the phenyl ring did not improve the cytotoxicity against both cell lines. However, compound 3d showed a better IC_50_ (178 μg/mL) than its analog 3a against HepG2 cell lines. Encouraged by this result, fluorine was replaced by chlorine at the same position on the phenyl ring in compounds 3d, 3e and 3f to afford compounds 3 g, 3 h and 3i. Surprisingly, compound 3 g and 3 h significantly inhibited the proliferations of HepG2 cell lines with IC_50_ of 124.46 and 120.62 μg/mL. The anticancer activities of these two compounds against HeLa cell lines were equally frustrating like the previous results. The possibility to change the position of chlorine from para to ortho in the phenyl ring was then explored. Interestingly, this modification proved detrimental, and compounds 3j, 3 k and 3 l displayed IC_50_ of 190 μg/mL.

### **Molecular modeling**

**Figure 2:**
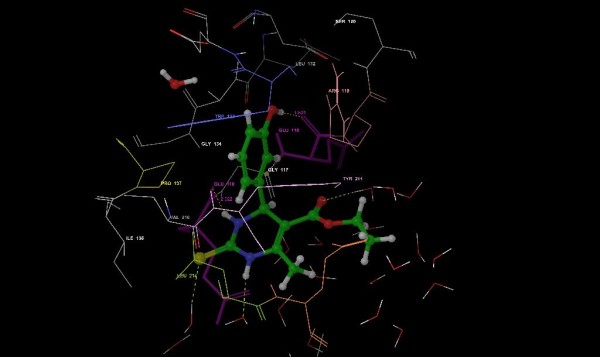
Compound 3c with Eg5 protein (1Q0B).

In order to further investigate the relationships between the virtual receptor-ligand binding interaction and physicochemical properties of the new compounds with their anticancer activity, molecular modeling studies were performed. Docking analysis revealed that hydrogen bond formation and hydrophobic interactions were the key factors affecting inhibitory action of the compounds. The Glu-116, Gly-117, Glu-118, Ar-119, Trp-127, Pro-137, Tyr-211 and Leu-214 of Eg5 protein were found to be directly interacting with the synthesized DHPMs. Most of the synthesized compounds showed hydrogen bonding interaction with Glu-116 as monastrol. However, the unsubstituted compounds, except 3c (Figure 2), lack the hydrogen-bonding interactions, which are weakly active to inhibit proliferation of cancer cell lines.

**Figure 3:**
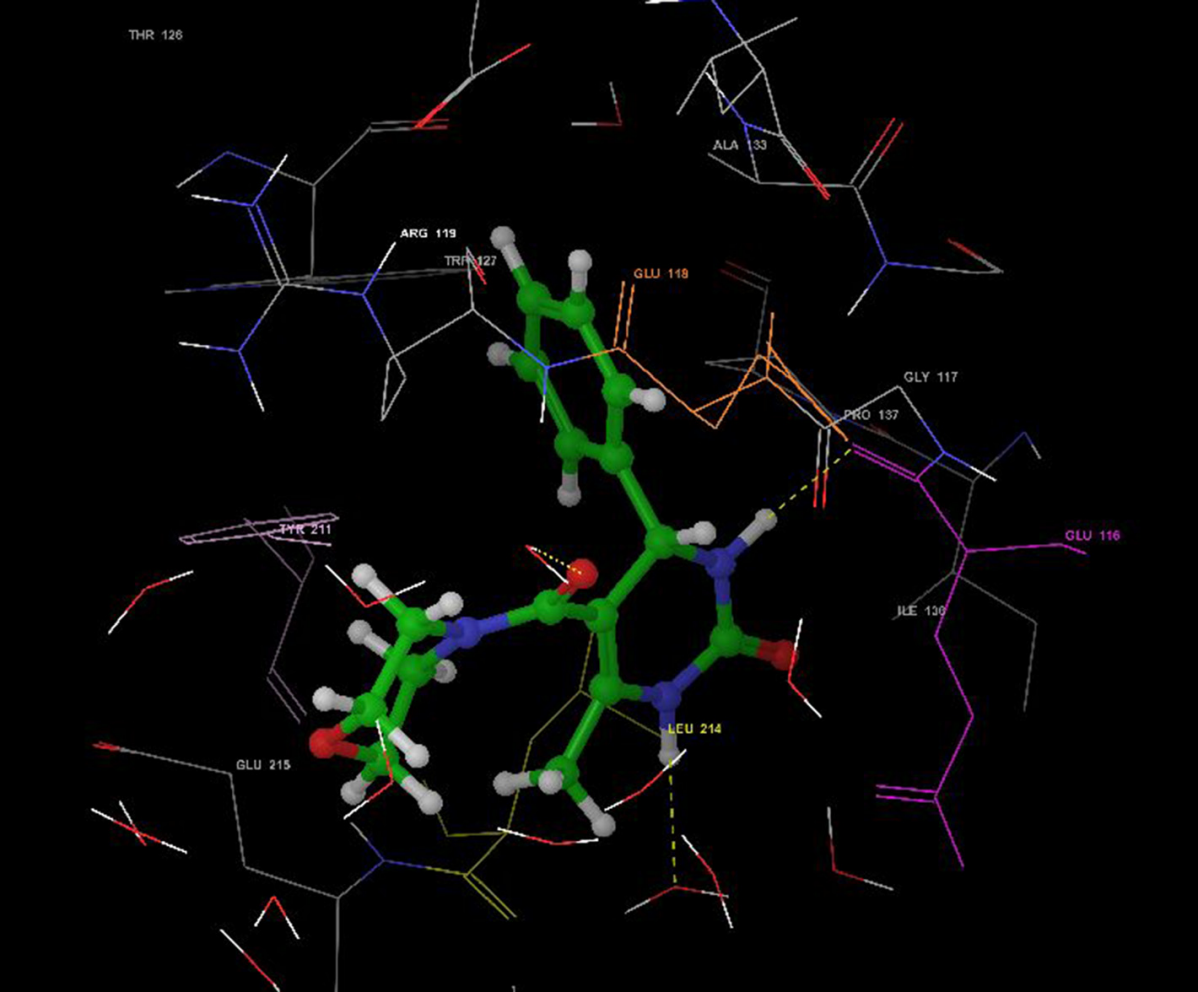
**Monastrol with Eg5 protein (1Q0B). **The yellow dotted lines represent hydrogen bonding interaction.

None of the compounds showed hydrogen bonding interaction with Glu-118. This may be attributed to the absence of hydrogen bond donors on the phenyl ring of DHPMs. Bioisosteric replacement of thiourea ‘S’ with urea ‘O’ in the synthesized compounds appeared to be oriented in similar fashion and retained similar interactions as monastrol. Co-crystallized S-monastrol when redocked in the active site of KSP attained a score of −9.78 kcal/mol. It displayed vital H-bonding interactions (−0.25495 kcal/mol) with the residues Glu-116 and Glu-118 (Figure 3).

The most active compounds, 3 h against HepG2 (Figure 1) and compound 3 g against HeLa, fitted best in the active site of Eg5 inhibitor protein and attained the score of −7.541568 kcal/mol and −8.667402 kcal/mol, respectively. It retained all the prime interactions to anchor well in the active sites of the receptor, except the missing hydrogen bonding with Glu-118. Moreover, hydrophobic interactions were observed to be involved in the binding of the most active compounds. All active compounds (compounds 3b, 3d, 3 g and 3 h) of the DHPM series were oriented in the active site of the protein in a way that places the aromatic ring into the pocket comprising the residues Glu-118, Arg-119, Trp-127, Pro-137 and Tyr-211. On the other hand, the ethyl group of S-monastrol and the cyclic amine substituents of DHPMs were almost pointed towards aqueous environment (Table 3).

**Table 3:** ***In silico *docking results**

**Compound**	**Docking score**^**a**^	**H-bonding with Glu-116**		**H-bonding with Glu-118**	
		**Distance (Å)**	**Energy (Kcal/mol)**	**Distance (Å)**	**Energy (Kcal/mol)**
Monastrol	−9.780843	2.022	−0.25495	1.800922	−0.25495
3a	−4.623578	1.794177	0	1.91532	0
3b	−4.099871	2.087333	0	2.437469	0
3c	−7.66649	2.184935	−0.155458	2.543947	0
3d	−3.518203	2.579143	−0.092150	2.579143	0
3e	−7.396385	2.231526	−0.123687	2.472871	0
3f	−3.336005	2.365419	−0.097571	2.477461	0
3 g	−8.667402	2.226021	−0.076754	2.496712	0
3 h	−7.541568	1.947079	−0.215195	2.240761	0
3i	−6.983773	2.04085	−0.226944	2.243988	0
3j	−7.419477	2.224107	−0.171504	2.25015	0
3 k	−6.988692	2.242662	−0.124171	2.197052	0
3 l	−6.8606	2.266054	−0.068286	2.199539	0

^a^Grid scoring from flexible docking (kcal/mol).

The virtual physicochemical parameters of the synthesized compounds were benchmarked against standard monastrol, and none of the descriptors were found to violate the optimal range of the parameters required for anticancer drugs. All the compounds validated Lipinski's rule of five, which extends the scope of performance in the *in vivo* studies. This shows the potential of the compounds to bind with the enzyme effectively and inhibit cell proliferation with minimized toxic effects. Optimal pharmacokinetic properties such as lipophilicity and absorption were established by the QLogP values and polar surface areas, which correlated with the number of hydrogen bond donors/acceptors (Table 4).

**Table 4:** ***In silico *physicochemical properties prediction**^**a**^

**Compound**	**Mol. Wt.**	**Log P**	**H donor**	**H acceptor**	**Rot. bonds**	**PSA (Å2)**
Monastrol	292.352	3.31	1	3.25	3	85.118
3a	285.345	3.406	0	3	1	78.312
3b	299.372	3.681	0	3	1	77.135
3c	301.344	2.533	0	4.7	1	86.394
3d	303.335	3.67	0	3	1	78.367
3e	319.335	2.818	0	3	1	77.133
3f	317.362	3.919	0	4.7	1	86.585
3 g	319.79	3.925	0	3	1	78.307
3 h	333.817	4.092	0	3	1	76.989
3i	335.789	3.083	0	4.7	1	86.586
3j	319.79	3.761	0	3	1	77.458
3 k	333.817	3.812	0	3	1	75.797
3 l	335.789	2.897	0	4.7	1	86.938

^a^QikProp v3.4 (Schrodinger-2010). H donor, hydrogen bond donor; H acceptor, hydrogen bond acceptor; Mol. Wt., molecular weight; Rot. bonds, number of rotational bonds; PSA, polar surface area in Å^2^.

## **Conclusion**

Eg5 represents a promising target, and designing Eg5 inhibitors would offer a novel approach to develop potent anticancer agents. A series of 12 novel monastrol analogs based on Biginelli reaction were synthesized with IC_50_ in the range of 120 to 398 μg/mL against HeLa and HepG2 cell lines. This new series consisted of pyrrolidine, piperidine and morpholine as privileged structures attached to the side chain of Biginelli dihydropyrimidinones through amide bond to improve the metabolic stability. SAR analysis and molecular modeling studies revealed that the positioning of a hydrogen bond donor/acceptor on the phenyl ring of the dihydropyrimidinone plays a crucial role in the inhibition of Eg5 enzyme to exhibit anticancer activity. Although the new compounds were found to have moderate to weak activity against cancer cell lines, the reported results are expected to contribute toward deeper insight into structure-activity relationship and could be helpful in further designing dihydropyrimidinones as potential anticancer agents.

## **Abbreviations**

DHPM: dihydropyrimidinones; HeLa: human hepatocellular carcinoma; HepG2: human epithelial carcinoma; MTT: 3-(4,5-dimethylthiazol-2-yl)-2,5-diphenyltetrazolium bromide; HCl: hydrochloric acid; TBTU- O: (Benzotriazol-1-yl)-N,N,N',N'- tetramethyluronium tetraflouroborate; DIPEA: diisopropylethylamine; IC_50_: concentration at 50% inhibition; SAR: structure activity relationship; Glu: glutamic acid; Gly: glycine; Arg: arginine; Trp: tryptophan; Tyr: tyrosine; Pro: proline; Leu: leucine.

## **Competing interests**

The authors declare that they have no competing interests.

## Supplementary Material

Additional file 1 Proton NMR spectrum of compound 1d. Click here for file

Additional file 2 Mass spectrum of compound 1d. Click here for file

Additional file 3 Carbon NMR spectrum of compound 3f. Click here for file

Additional file 4 Mass spectrum of compound 3f. Click here for file

Additional file 5 Proton NMR spectrum of compound 3 g. Click here for file

Additional file 6 Mass spectrum of compound 3 g. Click here for file

Additional file 7 Proton NMR spectrum of compound 3j. Click here for file

Additional file 8 Mass spectrum of compound 3j. Click here for file

## References

[B1] DeBonis S Skoufias DA Indorato R-L Liger F Marquet B Laggner C Benoît J Kozielski F Structure activity relationship of S-Trityl-L-Cysteine analogues as inhibitors of the human mitotic kinesin Eg5 J Med Chem 2008 51 1115 1125 18266314 10.1021/jm070606z

[B2] Matsuno K Sawada J Sugimoto M Ogo N Asai A Bis(hetero)aryl derivatives as unique kinesin spindle protein inhibitors Bioorg Med Chem Lett 2009 19 1058 1061 19167222 10.1016/j.bmcl.2009.01.018

[B3] Mayer TU Kapoor TM Haggarty SJ King RW Schreiber SL Mitchison TJ Small molecule inhibitor of mitotic spindle bipolarity identified in a phenotype-based screen Science 1999 286 971 974 10542155 10.1126/science.286.5441.971

[B4] Maliga Z Kapoor TM Mitchison T Evidence that monastrol is an allosteric inhibitor of the mitotic kinesin Eg5 J Chem Biol 2002 9 989 996 10.1016/s1074-5521(02)00212-0 12323373

[B5] Haque SA Hasaka TP Brooks AD Lobanov PV Baas PW Monastrol, a prototype anticancer drug that inhibits mitotic kinesin, induces rapid bursts of axonal outgrowth from cultured postmitotic neurons Cell Motil Cytoskeleton 2004 58 10 16 14983520 10.1002/cm.10176

[B6] Yoon SY Choi JE Huh JW Hwang O Lee HS Hong HN Kim D Monastrol, a selective inhibitor of mitotic kinesin Eg5, induces a distinctive growth profile of dendrites and axons in primary cortical neuron cultures Cell Motil Cytoskeleton 2005 60 181 190 15751098 10.1002/cm.20057

[B7] Klein E DeBonis S Thiede B Skoufias DA Kozielski F Lebeau L New Chemical tools for investigating human mitotic kinesin Eg5 Bioorg Med Chem 2007 15 6474 6488 17587586 10.1016/j.bmc.2007.06.016

[B8] Russowsky D Canto RFS Sanches SAA D'Oca MGM de Fátima A Pilli RA Kohn LK De Antônio MA Carvalho JE Synthesis and differential antiproliferative activity of Biginelli compounds against cancer cell lines: monastrol, oxo-monastrol and oxygenated analogues Bioorganic Chemistry 2006 34 173 182 16765411 10.1016/j.bioorg.2006.04.003

[B9] Horton D Bourne GT Smythe ML The combinatorial synthesis of bicyclic privileged structures or privileged substructures Chem Rev 2003 103 893 930 12630855 10.1021/cr020033s

[B10] Tu S Xiao-Tong Z Fang F Xiao-Jing Z Song-Lei Z Tuan-Jie L Da-Qing S Xiang-Shan W Shun-Jun J One pot synthesis of 3,4-dihydropyrimidin-2(1 H)- ones using boric acid as catalyst Tetrahedron Lett 2003 44 6153 6155

[B11] Wang Y Yu J Miao Z Chen R Bifunctional primary amine- thiourea-TfOH (BPAT·TfOH) as a chiral phase-transfer catalyst: the asymmetric synthesis of dihydropyrimidines Organic & Bimolecular Chemistry 2011 9 8 3050 3054 10.1039/c0ob01268h 21394354

[B12] Ma Y Qian C Wang L Yang M Lanthanoid triflate catalyzed Biginelli reaction- one-pot synthesis of dihydropyrimidinones under solvent-free conditions Journal of Organic Chemistry 2000 65 12 3864 3868 10864778 10.1021/jo9919052

[B13] Murata H Ishitani H Iwamoto M Synthesis of Biginelli dihydropyrimidinone derivatives with various substituents on aluminium- planted mesoporous silica catalyst Organic & Biomolecular Chemistry 2010 8 5 1202 1211 20165814 10.1039/b920821f

[B14] Litvić M Večenaj I Mikuldaš LZ Lovrić M Vinković V Filipan-Litvić M First application of hexaaquaaluminium(III) tetrafluoroborate as a mild, recyclable, non-hygroscopic acid catalyst in organic synthesis: a simple and efficient protocol for the multigram scale synthesis of 3,4-dihydropyrimidinones by Biginelli reaction Tetrahedron 2010 66 19 3463 3471

[B15] Barrow JC Nantermet PG Selnick HG Glass KL Rittle KE Gilbert KF Steele TG Homnick CF Freidinger RM Ransom RW Kling P Reiss D Broten TP Schorn TW Chang RSL Malley SSO Olah TV Ellis JD Barrish A Kassahun K Leppert P Nagarathnam D Forray C In vitro and in vivo evaluation of dihydropyrimidinone C-5 amides as potent and selective r1A receptor antagonists for the treatment of benign prostatic hyperplasia J Med Chem 2000 43 14 2703 2718 10893308 10.1021/jm990612y

[B16] Shutalev AD Aksionov AN Simple synthesis of 4-aryl-6-styryl-1,2,3,4- tetrahydropyrimidin-2-ones by the alkaline hydrolysis of Biginelli compounds Mendeleev Comm 2005 15 73 75

[B17] Steele TG Coburn CA Patane MA Bock MG Expedient synthesis of 5-unsubstituted-3,4-dihydropyrimidin-2(1 H)-ones Tetrahedron Lett 1998 39 9315 9318

[B18] Desai B Microwave-assisted solution phase synthesis of dihydropyrimidine C5 amides and esters Tetrahedron 2006 62 19 4651 4664

[B19] Movassagh B Balalaie S Shaygan P A new and efficient protocol for preparation of thiol esters from carboxylic acids and thiols in the presence of 2-(1 H- benzotriazole-1-yl)-1,1,3,3-tetramethyluronium tetraflouroborate (TBTU) ARKIVOC 2007 13 47 52

[B20] Balalaie S Mahdidoust M Eshaghi-Najafabadi R 2-(1 H-benzotriazole-1-yl)- 1,1,3,3-tetramethyluronium tetraflouroborate as an efficient coupling reagent for the amidation and phenylhydrazation of carboxylic acids at room temperature J Iran Chem Soc 2007 4 3 364 369

[B21] Mosmann T Rapid colorimetric assay for cellular growth and survival: application to proliferation and cytotoxicity assays J Immunol Methods 1983 65 1–2 55 63 6606682 10.1016/0022-1759(83)90303-4

[B22] Yan Y Sardana V Xu B Homnick C Halczenko W Buser CA Schaber M Hartman GD Huber HE Kuo LC Inhibition of a mitotic motor protein: where, how, and conformational consequences J Mol Biol 2004 335 547 554 14672662 10.1016/j.jmb.2003.10.074

[B23] Garcia-Saez I DeBonis S Lopez R Trucco F Rousseau B Thuery P Kozielski F Structure of human Eg5 in complex with a new monastrol-based inhibitor bound in the R configuration J Bio Chem 2007 282 13 9740 9747 17251189 10.1074/jbc.M608883200

[B24] Hu EH Sidler DR Dolling U-H Unprecedented catalytic three component one-pot condensation reaction: an efficient synthesis of 5-alkoxycarbonyl-4-aryl-3,4- dihydropyrimidin-2(1 H)-ones J Org Chem 1998 63 3454 3457

[B25] Dayal B Salen G Toome B Tint GS Shefer S Padia J Lithium hydroxide/aqueous methanol: mild reagent for the hydrolysis of bile acid methyl esters Steroid 1990 55 5 233 237 10.1016/0039-128x(90)90021-3 2163126

[B26] Kumar R Mittal A Ramachandran U Design and synthesis of 6-methyl-2-oxo- 1,2,3,4-tetrahydropyrimidine-5-carboxylic acid derivatives as PPAR activators Bioorg Med Chem 2007 17 16 4613 4618 10.1016/j.bmcl.2007.05.081 17574414

