# Supplementary material for: Monastrol mimic Biginelli dihydropyrimidinone derivatives: synthesis, cytotoxicity screening against HepG2 and HeLa cell lines and molecular modeling study
Source: Org Med Chem Lett. 2012 Jun 12;2:23. doi: 10.1186/2191-2858-2-23 (PMC3518143; doi:10.1186/2191-2858-2-23)
Supplement: Additional file 1 — Proton NMR spectrum of compound 1d. [file 2191-2858-2-23-S1.pdf]

DHPME-3.

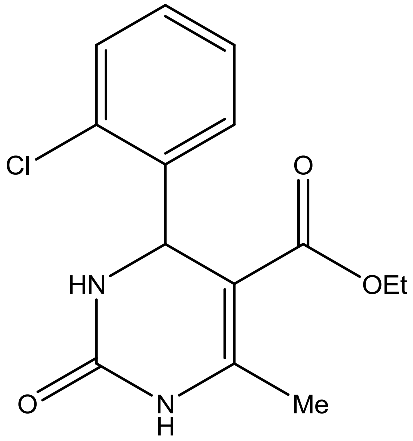

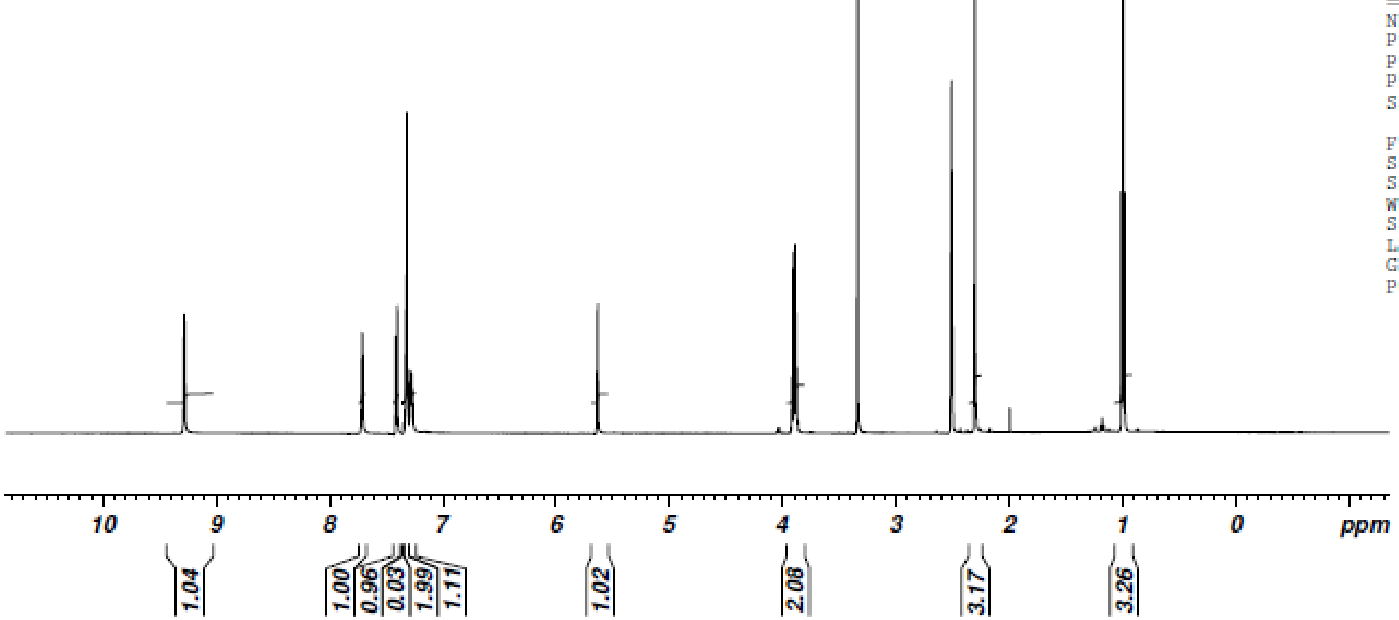

Current Data Parameters
NAME Mar18-2011
EXPNO 38
PROCNO 1

F2 - Acquisition Parameters
Date_ 20110319
Time 4.59
INSTRUM spect
PROBHD 5 mm PABBO BB-
PULPROG zg30
TD 32768
SOLVENT DMSO
NS 32
DS 2
SWH 10330.578 Hz
FIDRES 0.315264 Hz
AQ 1.5860212 sec
RG 203
DW 48.400 usec
DE 6.50 usec
TE 295.9 K
D1 1.00000000 sec
TD0 1

====== CHANNEL f1 ======
NUC1 1H
P1 10.65 usec
PL1 0.00 dB
PL1W 23.53637505 W
SFO1 500.1330885 MHz

F2 - Processing parameters
SI 32768
SF 500.1300000 MHz
WDW EM
SSB 0
LB 0.30 Hz
GB 0
PC 1.00
