# Supplementary figures and images for: Monastrol mimic Biginelli dihydropyrimidinone derivatives: synthesis, cytotoxicity screening against HepG2 and HeLa cell lines and molecular modeling study
Source: Org Med Chem Lett. 2012 Jun 12;2:23. doi: 10.1186/2191-2858-2-23 (PMC3518143; doi:10.1186/2191-2858-2-23)

DHPM-E-2\_002 #1 RT: 0.00 AV: 1 NL: 3.01E7  
T: + c ESI Full ms2 295.020 [50.000-300.000]

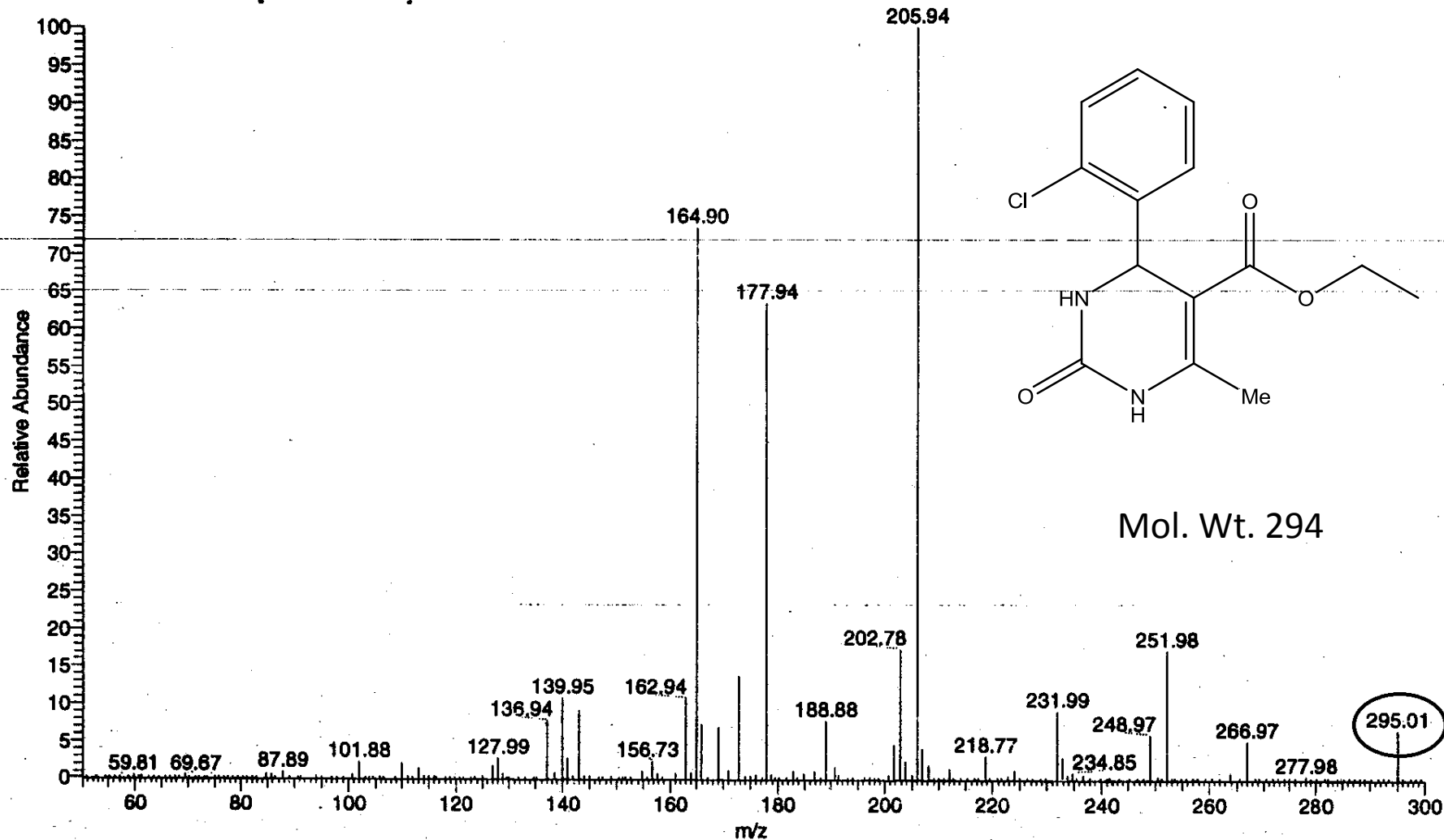

Supplement: Additional file 2 — Mass spectrum of compound 1d. [file 2191-2858-2-23-S2.pdf]

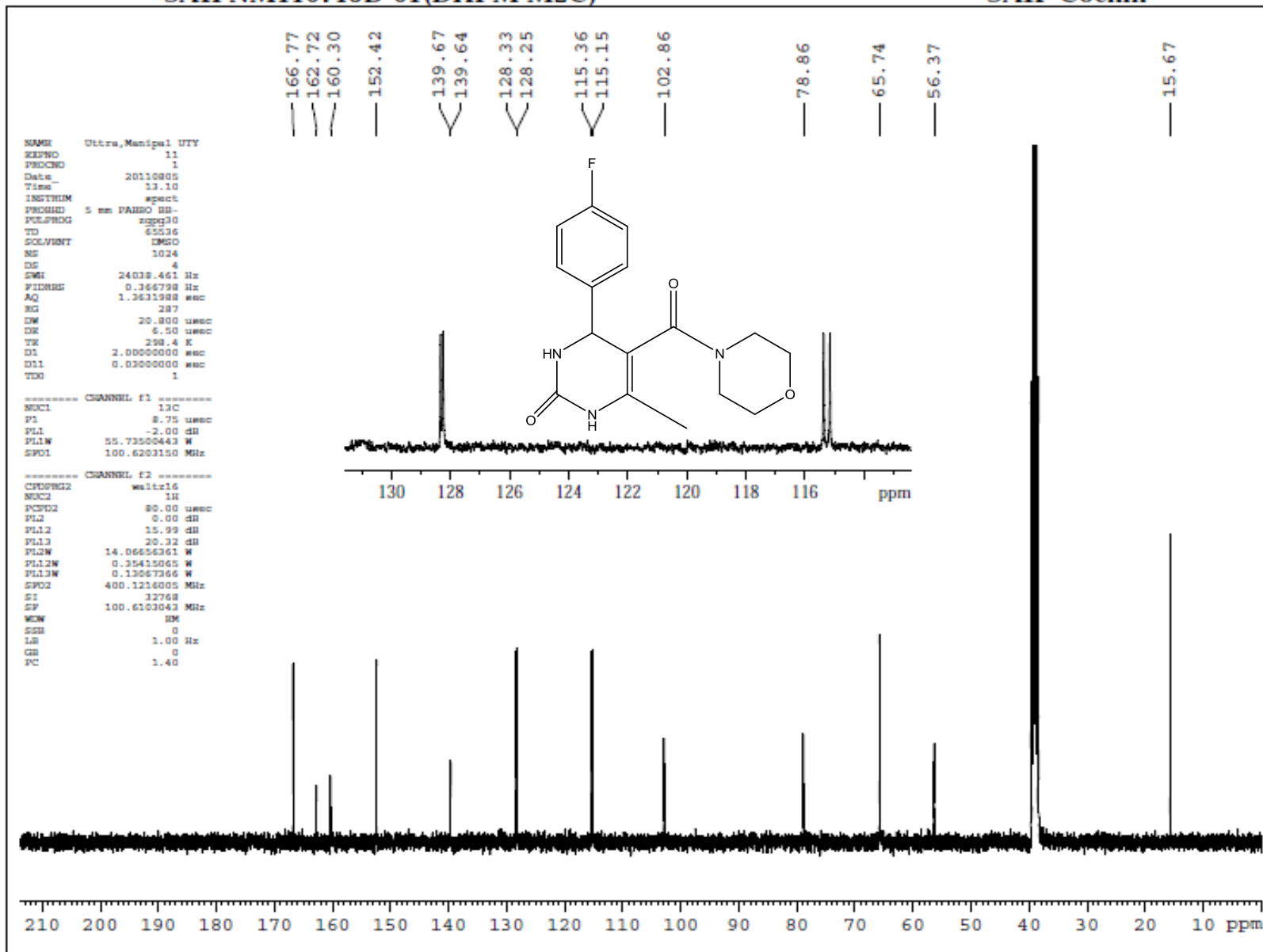

Supplement: Additional file 3 — Carbon NMR spectrum of compound 3f. [file 2191-2858-2-23-S3.pdf]

DHPM-M-3A\_110322164421 #1 RT: 0.01 AV: 1 NL: 1.03E8  
T: +c ESI Full ms2 320.000 [50.000-380.000]

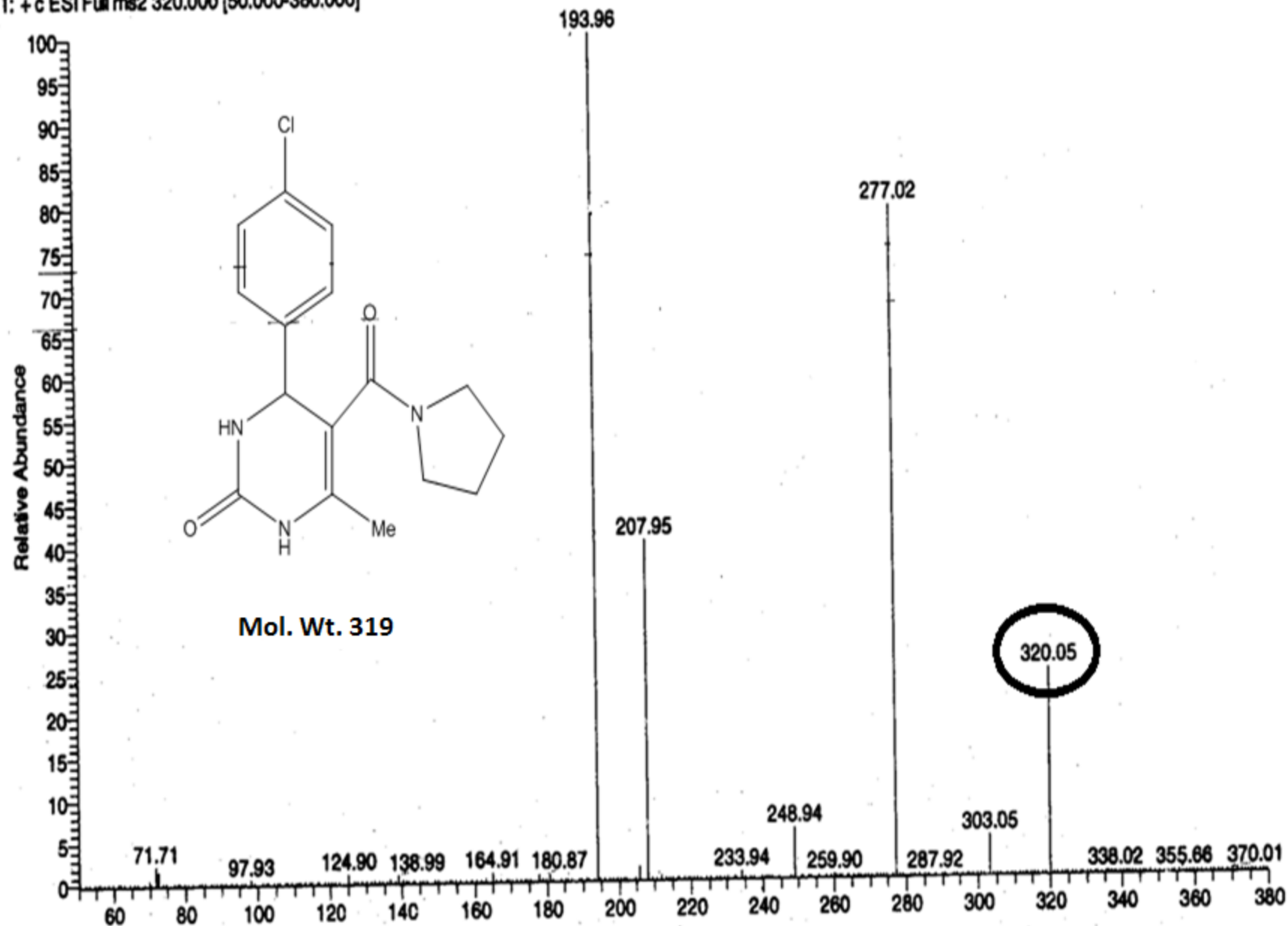

Supplement: Additional file 6 — Mass spectrum of compound 3 g. [file 2191-2858-2-23-S6.pdf]

Spectrum

Line#:1 R.Time:20.0(Scan#:2031)  
MassPeaks:199 BasePeak:70(799725)  
RawMode:Single 20.0(2031)  
BG Mode:None

intensity

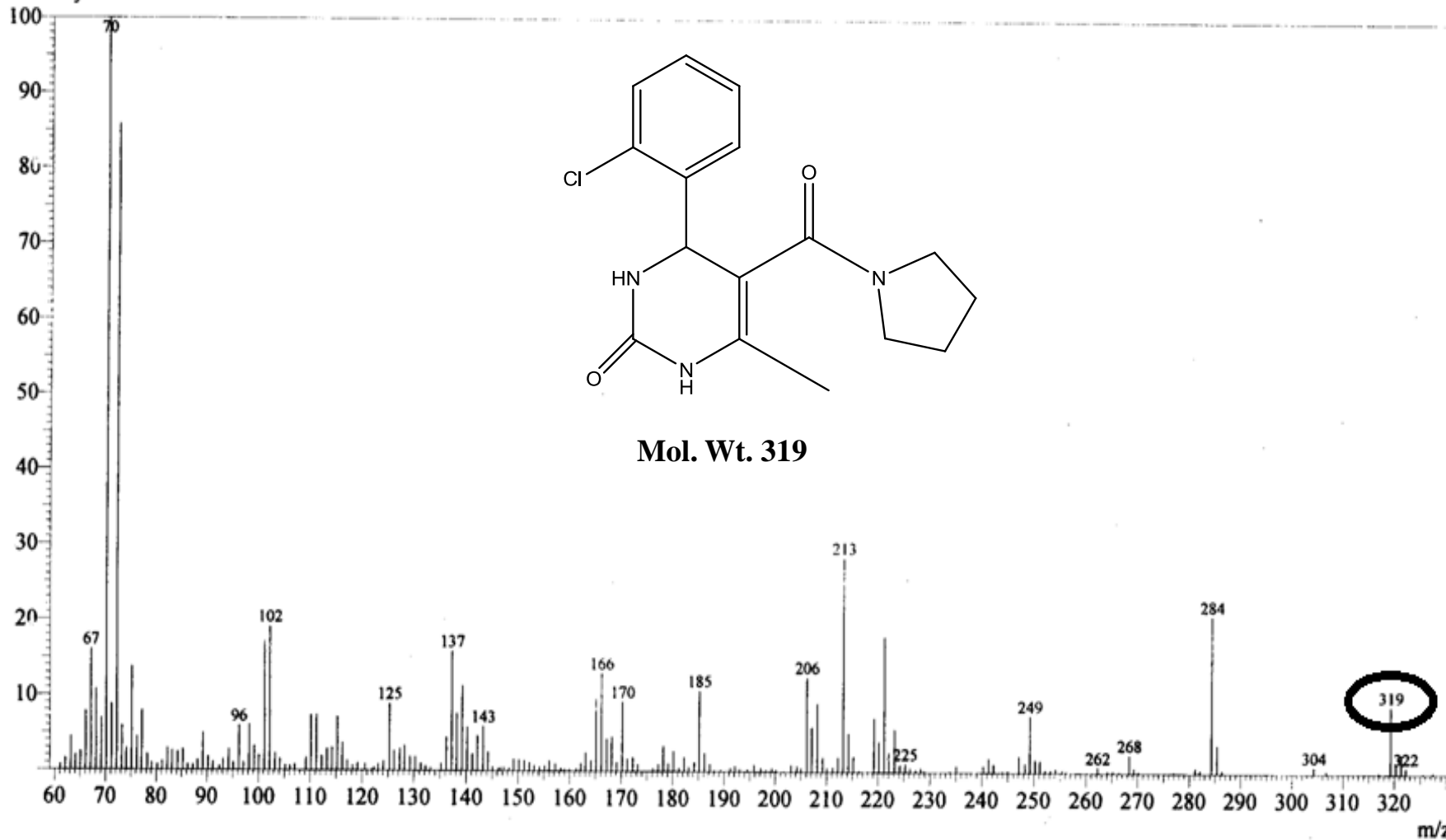

Supplement: Additional file 8 — Mass spectrum of compound 3j. [file 2191-2858-2-23-S8.pdf]
