# Supplementary material for: Monastrol mimic Biginelli dihydropyrimidinone derivatives: synthesis, cytotoxicity screening against HepG2 and HeLa cell lines and molecular modeling study
Source: Org Med Chem Lett. 2012 Jun 12;2:23. doi: 10.1186/2191-2858-2-23 (PMC3518143; doi:10.1186/2191-2858-2-23)
Supplement: Additional file 4 — Mass spectrum of compound 3f. [file 2191-2858-2-23-S4.pdf]

MANIPAL COLLEGE OF PHARMACEUTICAL SCIENCES, MANIPAL
Dept. of Pharma. Quality Assurance
GC-MS Analytical Report

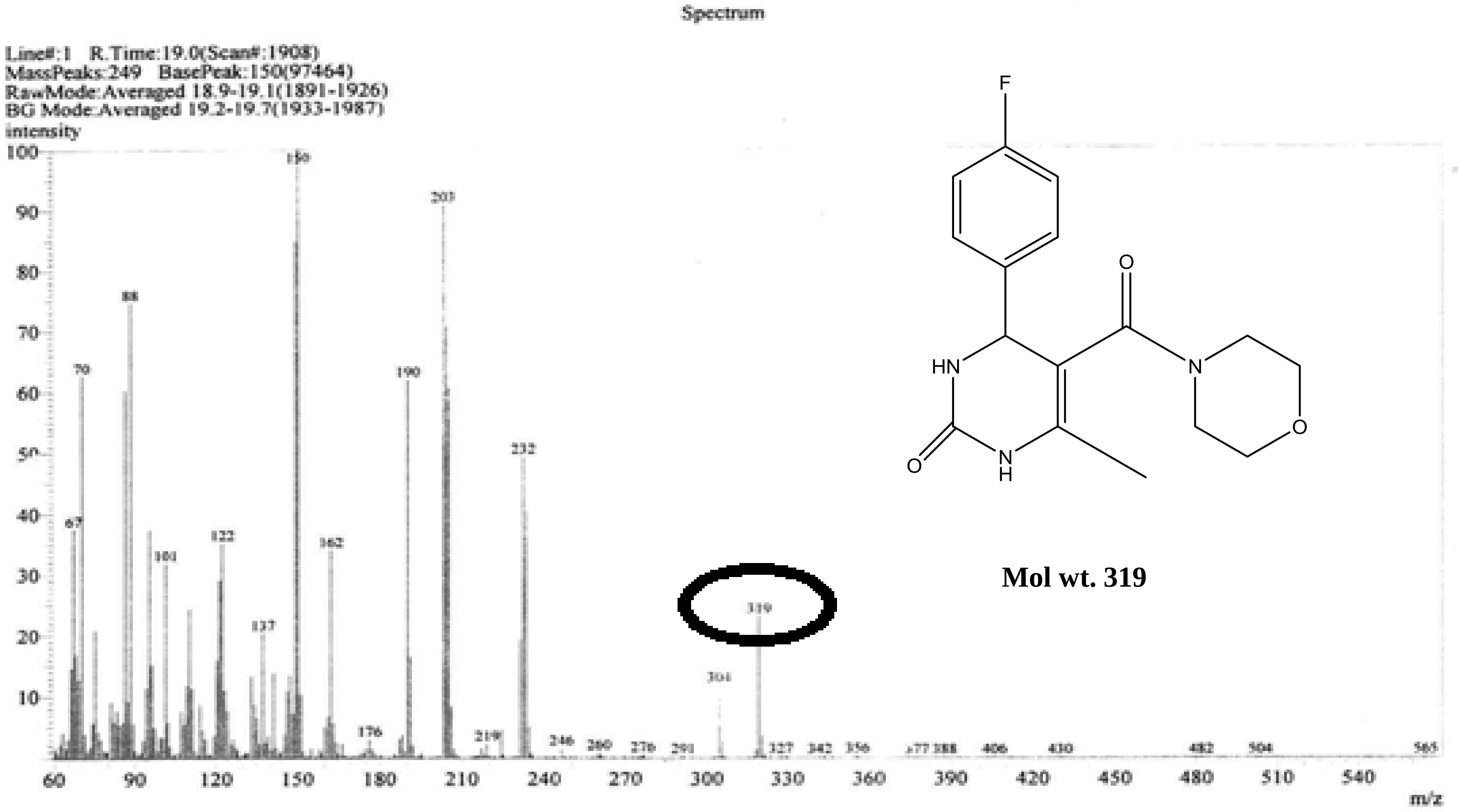

Mol wt. 319
