# Supplementary material for: Monastrol mimic Biginelli dihydropyrimidinone derivatives: synthesis, cytotoxicity screening against HepG2 and HeLa cell lines and molecular modeling study
Source: Org Med Chem Lett. 2012 Jun 12;2:23. doi: 10.1186/2191-2858-2-23 (PMC3518143; doi:10.1186/2191-2858-2-23)
Supplement: Additional file 5 — Proton NMR spectrum of compound 3 g. [file 2191-2858-2-23-S5.pdf]

vkmg-3a
PROTON CDCl3 {D:\May2011} niper 119

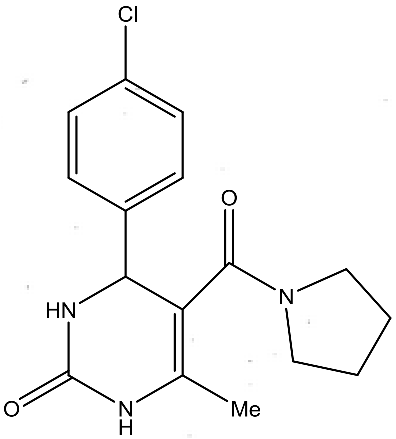

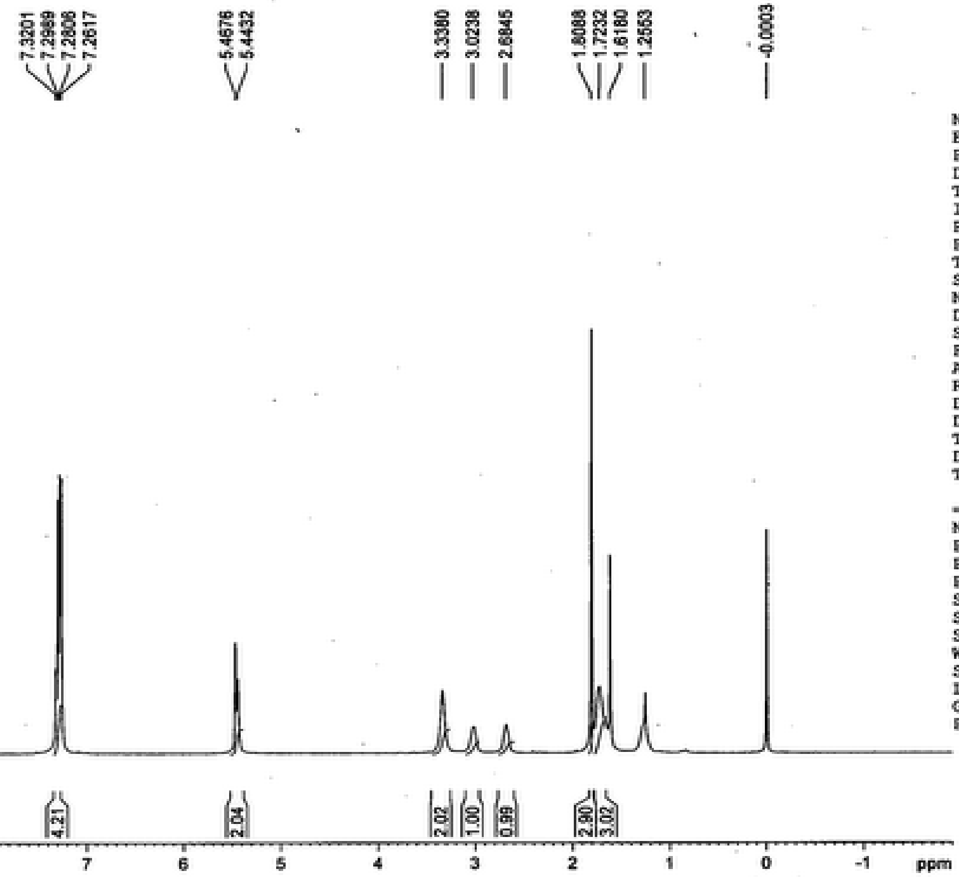

S.A.S. NAGAR

NAME	vkmg
EXPNO	10
PROCNO	1
Date_	20110505
Time	16.40
INSTRUM	spect
PROBHD	5 mm PABBO BB-
PULPROG	zg30
TD	65536
SOLVENT	CDCl3
NS	16
DS	2
SWH	8223.685 Hz
FIDRES	0.125483 Hz
AQ	3.9846387 sec
RG	203
DW	60.800 usec
DE	6.50 usec
TE	302.1 K
D1	1.00000000 sec
TD0	1

======== CHANNEL f1 ========

NUC1	1H
P1	12.20 usec
PL1	-2.00 dB
PL1W	14.80958652 W
SFO1	400.1324710 MHz
SI	32768
SF	400.1300052 MHz
WDW	EM
SSB	0
LB	0.30 Hz
GB	0
PC	1.40
