# Supplementary material for: Monastrol mimic Biginelli dihydropyrimidinone derivatives: synthesis, cytotoxicity screening against HepG2 and HeLa cell lines and molecular modeling study
Source: Org Med Chem Lett. 2012 Jun 12;2:23. doi: 10.1186/2191-2858-2-23 (PMC3518143; doi:10.1186/2191-2858-2-23)

SAIFNM110613A-02(DH PM M5A) SAIF Cochin

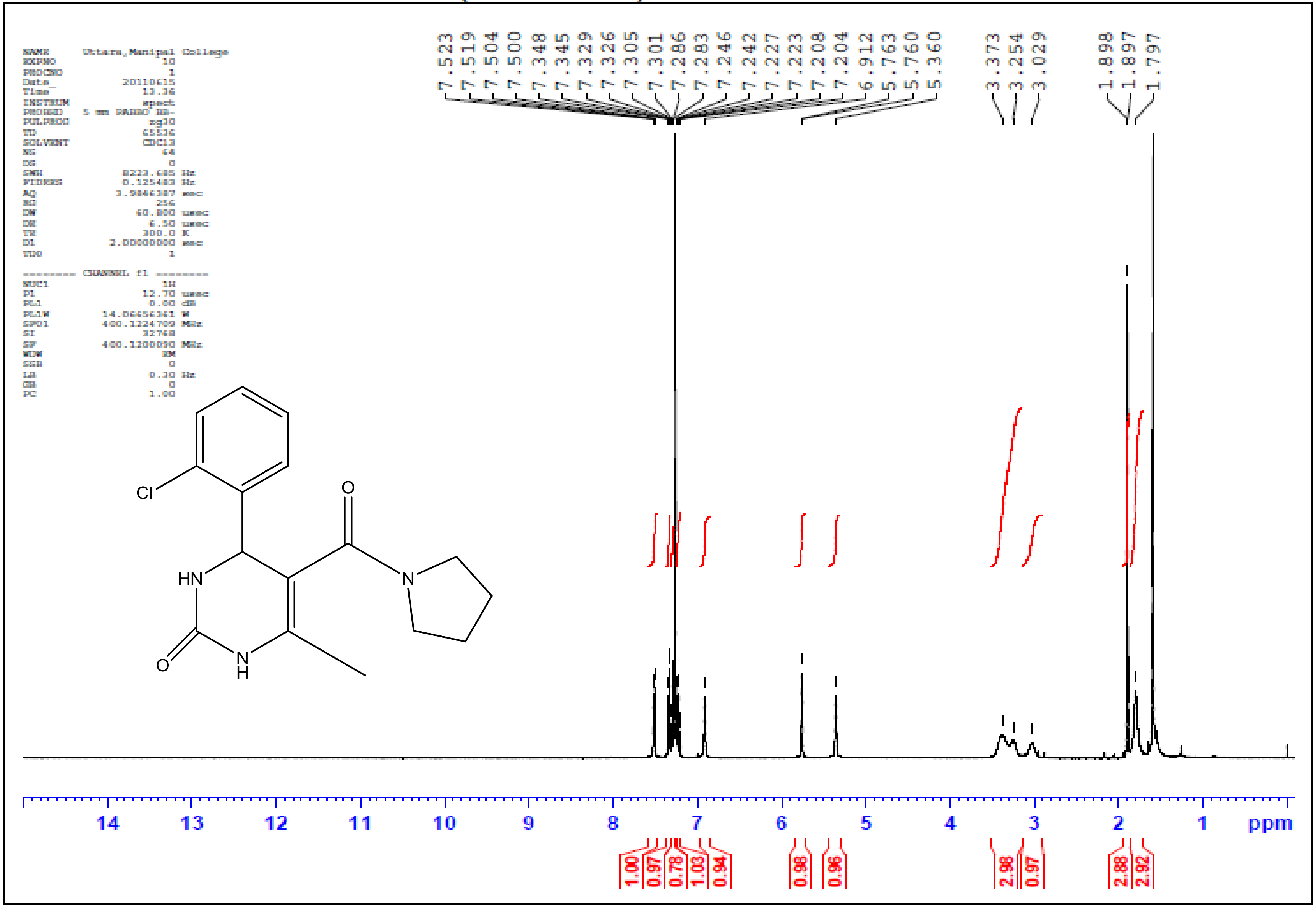

Supplement: Additional file 7 — Proton NMR spectrum of compound 3j. [file 2191-2858-2-23-S7.pdf]
